# Comparative Study on Composting and Vermicomposting to Improve Physicochemical Properties of Digestate with the Addition of Kitchen Waste^§^

**DOI:** 10.17113/ftb.63.02.25.8904

**Published:** 2025-06

**Authors:** Ze Sen Tan, M. Devendran Manogaran, Rashid Shamsuddin, Mohd Hakimi, Lee Wen Looi, Kai Tong Woo, Chin Seng Liew, Lailatul Qomariyah

**Affiliations:** 1 Chemical Engineering Department, Faculty of Engineering, Universiti Teknologi PETRONAS, 32610 Seri Iskandar, Perak, Malaysia; 2 Centre of Carbon Capture, Utilisation and Storage, Institute of Sustainable Energy and Resources, Universiti Teknologi PETRONAS, 32610 Seri Iskandar, Perak, Malaysia; 3 Department of Chemical Engineering, Faculty of Engineering, Islamic University of Madinah, 42311 Madinah, Saudi Arabia; 4 Department of Industrial Chemical Engineering, Institut Teknologi Sepuluh Nopember, 60111 Surabaya, Indonesia

**Keywords:** conventional composting, vermicomposting, chicken manure digestate, kitchen waste, improvement of physicochemical properties, macronutrient enrichment

## Abstract

**Research background:**

The rapid growth of the Malaysian population has led to an increase in kitchen waste, especially inedible organic kitchen waste, which is generally disposed of in landfills and pollutes the environment. Apart from this, the increasing demand for chicken products in Malaysia has led to a significant increase in chicken manure production. As anaerobic digestion continues to be explored, there are concerns about the utilization of the digestate from chicken manure. Therefore, this study addresses the challenge of treating kitchen waste and chicken manure digestate in Malaysia by investigating the effectiveness of composting and vermicomposting methods through comparative analysis. By integrating kitchen waste, particularly spent coffee grounds, bone waste and used kitchen towels, this study aims to improve the imbalanced physicochemical properties of digestate from chicken manure.

**Experimental approach:**

Before composting, the kitchen waste and chicken manure digestate were characterised to determine the initial physicochemical properties. Four composting setups comprising the substances were established to study the physical appearance, temperature and pH profile, the increase in nitrogen, phosphorus and potassium content, and the mass reduction of the final compost after 50 days of composting.

**Results and conclusions:**

The vermicompost with kitchen waste additives showed a significant nutrient improvement with an NPK mass ratio of 1:3.57:6.58 and a lower moisture mass fraction of 48.92 %, which requires the shortest maturation time (20 days) and the highest mass reduction (55.11 %).

**Novelty and scientific contribution:**

The novelty of this research is the valorisation of organic kitchen waste and chicken manure digestate as biofertiliser. The end result is achieved by promoting a sustainable alternative to exploit kitchen waste instead of the traditional approach of landfilling waste. At the same time the problem of digestate is addressed, particularly its unbalanced physicochemical properties, especially its macronutrients, pH and moisture content. In contrast to previous studies, this work investigates the effectiveness of both conventional composting and vermicomposting with the incorporation of organic kitchen waste, namely spent coffee grounds, bone meal and used kitchen towels, to improve the physicochemical properties of digestate.

## INTRODUCTION

In Malaysia, the problem of kitchen waste has worsened over the years due to population growth and Malaysian food ethics. An average of 17 007 tonnes of kitchen waste is generated daily, of which 12 926 tonnes is inedible and 4081 tonnes is edible kitchen waste, with over 90 % of kitchen waste being biodegradable (*1*, *2*). Kitchen waste is in direct conflict with the objectives of the Sustainable Development Goals (SDG), which aim to address global food loss and waste through responsible food consumption and production (*3*). Unfortunately, approx. 80 % of this organic waste ends up in landfills, increasing greenhouse gas emissions and posing a threat of soil and groundwater contamination due to leaching of nutrients (*1*). The approach of landfilling waste leads to missed opportunities to reintroduce value-added components back into the economy, resulting in higher capital investment requirements to develop new resources (*4*). Among the various types of kitchen waste, spent coffee grounds, bone waste, banana peels and used kitchen towels are among the organic kitchen waste rich in nitrogen, phosphorus, potassium and carbon, respectively, representing a mixture of inedible and unavoidable materials that are abundant in Malaysian households with high consumption.

The poultry sector in Malaysia has experienced significant growth in recent decades, driven by the increasing demand for chicken products. In 2022, consumption of chicken meat per capita was approx. 50.1 kg, resulting in a daily production of approx. 26 424 tonnes of chicken manure (*5*). Due to the high moisture content and biodegradability of chicken manure, anaerobic digestion has emerged as a common technology for its treatment (*6*). In the absence of oxygen, anaerobic digestion involves the hydrolysis of organic matter by microorganisms, followed by acidogenesis and methanogenesis to convert the intermediate products into biogas. Despite the low production of biogas per batch compared to the huge amount of nutrient-rich chicken manure digestate, anaerobic digestion continues to receive considerable research attention from academia and industry (*7*). As an underexploited by-product rich in organic and inorganic nutrients, the full potential of chicken manure digestate has yet to be revitalised by the agricultural industry. Although the direct application of chicken manure digestate as a biofertiliser is an alternative in agriculture to reduce the dependence on synthetic fertilisers (*8*), it is not suitable for plant uptake due to its sludgy texture and high moisture content. This poses the risk of leaching macronutrients such as NPKC, which are particularly important for optimal fertilisation. This has led to the insufficient nutrient supply to plants, resulting in stunted plant growth and overall poor vegetative development (*9*-*12*).

To improve the quality and stability of chicken manure digestate as a biofertiliser, composting is an evergreen and economically viable approach for households and the agricultural industry due to its cost-effectiveness and scalability to revitalise organic waste (*5*, *13*). Studies confirm its efficacy in enriching the environment, reducing landfilling waste and greenhouse gas emissions and promoting living landscapes (*8*, *14*). Chicken manure digestate can either be composted with other nutrient-rich organic materials or vermicomposted with the help of earthworms to improve the quality of the end product, the compost (*15*). As a subbranch of composting, vermicomposting has gained recognition as an environmentally friendly waste management approach, mainly because it saves time compared to conventional composting (*16*). Vermicompost contains high amount of nutrients (N, P, K, humic and fulvic acid) in a plant-accessible form, improved microbial activity and water retention (*16*-*18*).

Although researchers have repeatedly explored the synergies between chicken manure digestate and other organic matter to improve the composting process and its physicochemical quality (*17*, *19*), little attention has been paid to the formulation of organic kitchen waste and chicken manure digestate in previous studies. Additionally, the comparative analysis between composting and vermicomposting techniques with and without the incorporation of organic kitchen waste and their effects on improving the physicochemical properties of chicken manure digestate has not been conducted. This study explicitly aims to fill this research gap by systematically comparing composting and vermicomposting as techniques to improve the physicochemical properties of chicken manure digestate. The study focuses on the effectiveness of these methods in combination with certain kitchen wastes such as spent coffee grounds, bone waste, banana peels and used kitchen towels. By emphasising the strengths and limitations of each approach, this research highlights their potential for wider application in waste management and agricultural practices.

These kitchen wastes have been shown to be biodegradable, so they can potentially improve texture and nutrient content of chicken manure digestate that is composted, increasing the value of the compost produced while supporting the circular economy model, as discussed by Hashim *et al*. (*1*). Therefore, this research focuses on proposing a sustainable alternative to the conventional practice of landfilling kitchen waste while addressing the problem of unbalanced physicochemical properties of chicken manure digestate as a biofertiliser. It studies the effectiveness of composting and vermicomposting to improve physicochemical properties of chicken manure digestate with organic kitchen waste as organic additives. Due to the ease of implementation, this study also serves as a basis for future commercialisation on an industrial scale. These efforts are in line with the United Nations Sustainable Development Goals (SDGs) of affordable and clean energy, sustainable cities and communities, responsible consumption and production, and climate action (*3*).

## MATERIALS AND METHODS

### Sample preparation

The chicken manure digestate was obtained from a biogas pilot-scale operation in Manjung district, Perak, Malaysia. The liquid and solid fractions of the digestate were separated using a large-volume centrifuge, model Z513K (Hermle, Gosheim, Germany) at 1036*g* for 40 min per batch. The liquid fraction was kept to moisturise the compost, while the solid fraction was used as composting material.

Spent Arabica coffee grounds (Soo Hup Seng Trading Co, Penang, Malaysia), Cavendish banana peel (Simple Farm Group, Johor, Malaysia) and used kitchen towels (Premier, Bangalore, India) were collected from the local cafeteria and dried overnight at 105 °C to remove moisture. Waste chicken bones were collected from a local cafeteria at Universiti Teknologi PETRONAS, Seri Iskandar, Perak, Malaysia. The bones were thoroughly washed to remove the residual meat from the surface, followed by 6 h of boiling. The boiled bones were dried overnight at 105 °C and ground into a fine powder.

### Characterisation of organic substances

The raw materials were characterised to determine the physicochemical properties and the analysis was repeated on compost samples to evaluate the maturity and degree of improvement of nutrients. The Elementar Vario Micro Cube carbon, hydrogen, nitrogen and sulphur analyser (Elementar, Frankfurt, Germany) was used to determine the C and N content, requiring 2.5 g of each sample in dried form. The C and N content of the samples was determined through combustion in an oxygen-rich environment, converting the elements into measurable gases, which were quantified using the built-in thermal conductivity detector. The K content of the samples was determined using the Shimadzu AA6800 atomic absorption spectroscopy analyser (Shimadzu, Kyoto, Japan). Liquid samples were prepared and diluted accordingly with a dilution factor of 100 to facilitate vaporisation, as the concentration of K was measured by the absorption of light at *λ*=766.5 nm. The moisture content of each sample was determined using the Mettler Toledo moisture analyser (Mettler Toledo, Greifensee, Switzerland), which combusted the samples and calculated losses of the moisture content as the mass difference. Hanna Direct Soil Measurement pH portable meter (Hanna Instruments, Woonsocket, RI, USA) was used to determine the pH value of the waste samples and the compost media.

The P content was determined using the Hach method 8190 (*20*) and the Hach DR3900 spectrophotometer (Hach, Ames, IA, USA). A volume of 5 mL of diluted liquid samples was added to the total phosphorus test vials (Hach), followed by 0.5 g of potassium persulfate powder (Hach) per each vial. The vials were shaken well before the digestion in DRB200 reactor (Hach), which was preheated to 150 °C for 30 min. Then the vials were removed from the reactor and cooled to room temperature. A volume of 2 mL of 1.54 M sodium hydroxide standard solution (Hach) was mixed into each vial, followed by adjusting the absorbance reading to zero. A mass of 0.5 g of PhosVer 3 powder (Hach) was added to each vial and shaken thoroughly until the colour change was observed. After a 2-minute reaction time, the vials were placed back into the spectrophotometer and the absorbance was read at *λ*=880 nm.

### Composting and vermicomposting

The required mass of each material for effective composting was calculated based on the nutrient content of each raw material. The study focused on the chicken manure digestate as the limiting reactant. Therefore, the mass of the other organic additives should not exceed that of the chicken manure digestate per setup, with the total initial compost mixture, *m*_1_, fixed at 1.5 kg. The predicted mass fraction (%) of each nutrient element in the final compost was calculated using the following equation:


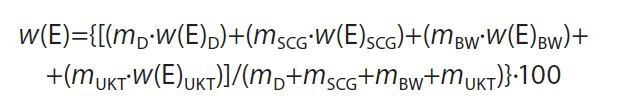
 /1/

where *w*(E) is the mass fraction of nutrient in the final compost, *w*(E)_D_ is the mass fraction of nutrient element in chicken manure digestate, *w*(E)_SCG_ is the mass fraction of nutrient element of spent coffee grounds, *w*(E)_BW_ is the mass fraction of nutrient element in bone waste, *w*(E)_UKT_ is the mass fraction of nutrient element in used kitchen towels, *m*_D_ is the mass of chicken manure digestate, *m*_SCG_ is the mass of spent coffee grounds, *m*_BW_ is the mass of bone waste and *m*_UKT_ is the mass of used kitchen towels. The compost samples were adjusted to a C/N ratio of 10, which was chosen to ensure a sufficient supply of C to be utilised by the bacteria and earthworms as they decompose the organic matter, as well as to regulate the temperature and pH value of the composting system.

The organic materials were mashed into small pieces and mixed well to achieve the ideal particle size range of 5 to 20 cm for enhanced aeration and moisture retention during composting (*21*). The composting activity was carried out using four setups of 15 L in covered black plastic containers (dimensions 19 cm×10.23 cm×22.5 cm; Eco-Shop, Kuala Lumpur, Malaysia) with 20 aeration holes. The compost setups were wrapped with green PVC garden netting (Baba Gardening, Penang, Malaysia) to protect them from pest invasion. The initial feedstock for each composting setup is shown in Table S1. For the vermicomposting setups (setups B and D), 100 *Eisenia fetida* earthworms (Earth Worm Enterprise, Perak, Malaysia) were placed in the setup to decompose the organic matter. *Eisenia fetida* is preferred because it promotes a less time-consuming process due to high rate of consumption and digestion of organic substances and at the same time has a greater tolerance to different environmental conditions and a high reproduction rate (*22*). A mass of 1.5 kg of gardening soil (Baba Gardening, Penang, Malaysia) was added to the vermicomposting at a gardening soil to compost mixture ratio of 1:1 to serve as the earthworm bedding.

The moisture content and mass of the initial compost were recorded before the beginning of composting to ensure between 45 and 60 % of moisture for optimal aerobic conditions and promoting the growth of microbes (*13*, *15*). The pH and temperature of each setup were recorded in triplicate at 4:00 pm every other day. This was to ensure that the pH value remains within the range of 4.5 to 8.5 and to monitor the temperature progression, which includes mesophilic phase (25 to 40 °C), thermophilic or curing phase (>40 °C) and psychrophilic phase (−10 to 20 °C) (*23*-*25*). After the readings were taken, the composter was watered with 5 mL of digestate liquid fraction and mixed to ensure adequate aeration and effective aerobic decomposition (*26*). Any physical observation on the pile and earthworms was noted as well.

### Improvement of the physicochemical properties of compost

After reaching day 50 of the composting process, the physicochemical properties of the compost were recorded and evaluated, namely the colour and texture of each compost product, as well as the pH, moisture content, mass yield of the compost and nitrogen, phosphorus, potassium and carbon (NPKC) content. The mass yield (%) was then calculated using the following equation (*26*):


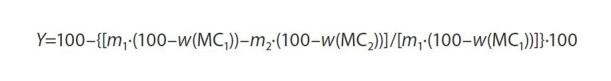
 /2/

where *m*_1_ is the mass of the initial compost mixture in kg, *m*_2_ is the final mass of mature compost in kg, *w*(MC_1_) and *w*(MC_2_) are the mass fractions (in %) of moisture in the initial and final compost mixture, respectively. On the other hand, the relative enrichment (RE/%) of the elements in the final compost was evaluated by systematic comparisons of the NPKC content of the final compost produced from different setups. These comparisons were made using the following equation:


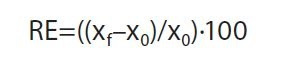
 /3/

where x_0_ and x_f_ are the concentrations of elements in feedstock and compost, respectively. RE>0 represents the potential enrichment of a particular element, while RE<0 indicates the loss of the element due to volatilisation (*8*, *26*). One-way analysis of variance (ANOVA) and least significant difference (LSD) were observed when the physicochemical enhancement and RE were significant at p<0.05 LSD using Microsoft Excel, v. 2019 (Microsoft Corporation, Washington DC, USA).

## RESULTS AND DISCUSSION

### Physicochemical properties of organic substance

The original proposal to use kitchen waste as an organic additive is based on extensive literature studies that point to the abundance of specific nutrients in the selected kitchen waste. These additives, namely spent coffee grounds for N, bone waste for P, banana peel for K and used kitchen towels as a C bulking agent, aim to improve the nutrient profile of chicken manure digestate for use as fertiliser. To test these proposals, each additive was characterised using different analytical techniques, as shown in Table 1 (*8*, *13*, *27*-*33*).

**Table 1:** Physicochemical properties of the organic substances (*8*, *13*, *26*-*32*)

Property		Chicken manure digestate	Spent coffee grounds	Bone waste	Banana peels	Used kitchen towels
*w*(C)/%	C_A_	(35.0±1.2)	(50.0±0.6)	(33.3±2.7)	(41.3±0.7)	(39.9±0.1)
	C_L_	36.0 (*8*)	46.2 (*27*)	43.1 (*29*)	43.7 (*31*)	N/A
*w*(N)/%	N_A_	(4.87±0.08)	(4.54±0.05)	(5.7±0.3)	(3.27±0.07)	(2.57±0.02)
	N_L_	4.5 (*8*)	2.4 (*27*)	15.7 (*29*)	1.5 (*31*)	N/A
*ξ*_C,N_	C/N_A_	(7.2±0.2)	(11.0±0.2)	(5.8±0.3)	(11.9±0.1)	(15.5±0.1)
	C/N_L_	8.0 (*8*)	19.5 (*27*)	22.3 (*29*)	29.9 (*31*)	N/A
*w(*P)/%	P_A_	(2.1±0.3)	(2.5±0.2)	(22.26±0.03)	(1.40±0.08)	(0.4±0.1)
	P_L_	1.7 (*8*)	0.9 (*27*)	41.0 (*29*)	1.6 (*31*)	N/A
*w*(K)/%	K_A_	(5.1±0.1)	(6.54±0.06)	(4.6±0.1)	(5.26±0.08)	(1.4±0.1)
	K_L_	2.1 (*8*)	3.7 (*27*)	0.03 (*29*)	7.8 (*32*)	N/A
*w*(moisture)/%	MC_A_	(83.0±0.9)	(62.3±1.1)	(7.7±0.3)	(10.9±0.5)	(67.0±0.7)
	MC_L_	67.5 (*13*)	61.0 (*28*)	63.8 (*30*)	89.1 (*33*)	N/A

Literature values are not presented with standard deviation. X_A_=elemental composition obtained through characterisation, X_L_=elemental composition obtained from literature study, N/A=not available in the literature. Results are expressed as mean value±standard deviation, *N*=3

While the actual and literature values for the C, N and P mass fractions in chicken manure digestate were consistent, a notable variance was observed in K mass fraction, which exceeded the literature value by more than double. This variance may be attributed to the fluctuating K mass fraction in the chicken manure used for anaerobic digestion, which is influenced by nutrient variations in the chicken farm fodder (*34*). The solubility of K ions in water and the freshness of the digestate samples during testing can also significantly affect the resulting K mass fraction (*8*, *26*).

Based on the results in Table 1, spent coffee grounds had the highest C mass fraction (over 50 %), followed by banana peel (41.3 %) and used kitchen towels (39.87 %). In contrast, bone waste proved to be the additive with the highest N mass fraction (5.74 %), surpassing spent coffee grounds (4.54 %). When analysing the C/N mass ratio, an important indicator of nutrient balance during composting, it was found that used kitchen towels had the highest C/N mass ratio due to their lower N content. Although the P mass fraction of bone waste was lower (22.26 %) than the literature value (40.99 %), it remained the additive with the highest P mass fraction. Spent coffee grounds contained the highest K mass fraction (6.54 %), followed by banana peel (5.26 %), bone waste (4.55 %) and used kitchen towels (3.14 %). This is in agreement with previous findings emphasising the K mass fraction in spent coffee grounds compared to other nutrients (*27*). Chicken manure digestate had the highest moisture mass fraction, highlighting the importance of incorporating additives with lower moisture content to improve the aerobic composting environment (*13*). Based on the characterisation results, spent coffee grounds (highest C and K mass fraction), bone waste (highest N and P mass fraction) and used kitchen towels (highest C/N mass ratio) were selected to improve chicken manure digestate nutrients, while banana peels were excluded from the composting.

Variations between the characterisation values and the literature data were attributed to several factors. For example, the variance in the nutrient content of spent coffee grounds can be attributed to the variety of coffee beans, roasting methods and brewing techniques before disposal (*27*). The observed variation in bone waste values may be due to differences in the origin of the chickens, fodder used during the chickens’ growth and the methods used to prepare bone waste samples, resulting in N mass fraction variations (*35*, *36*). In the case of banana peels, factors such as type, freshness and analytical techniques contributed to differences in nutrient content (*31*, *32*). Although no comparisons could be made for used kitchen towels due to the lack of previous work, this study provides a basis for future research investigation of their nutrient content in detail.

### Physical observation of composting process

**Fig. S1:**
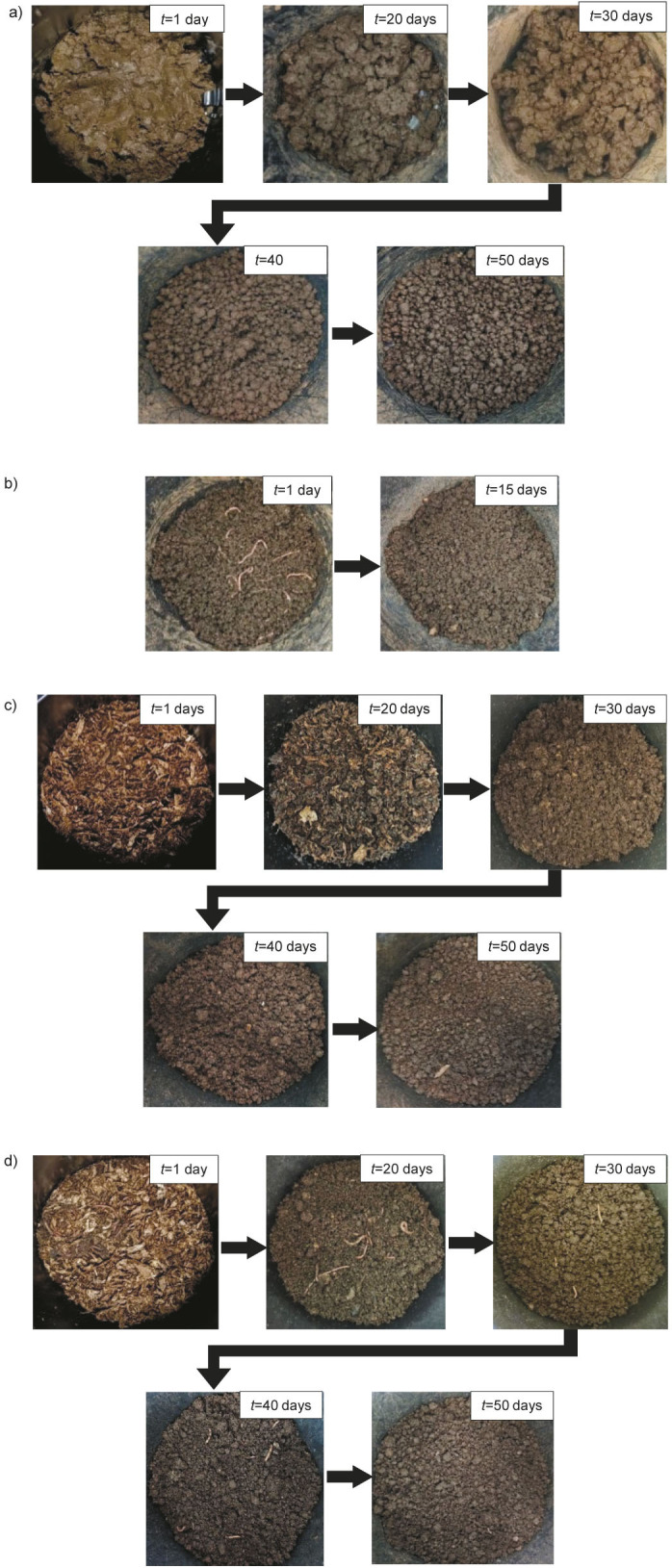
Physical observations of: a) setup A, in which the moisture content was reduced during 50 days of composting, b) setup B during 15 days of composting (setup was abandoned after 15 days due to the death of the earthworms), c) setup C during 50 days of composting, in which gradual decomposition of organic matter was observed, and d) setup D during 50 days of composting, in which rapid decomposition of organic matter was observed as visual inspection remained constant after *t*=20 days. Setup: A=pure chicken manure digestate, B=vermicomposted pure chicken manure digestate, C=mixture of chicken manure digestate and kitchen waste and D=vermicomposted mixture of chicken manure digestate and kitchen waste

The physical changes in each composting setup were recorded, as shown in Fig. S1. Four composting setups were prepared to study the efficiency of composting and vermicomposting, with and without organic kitchen waste additives, in improving the NPKC content of chicken manure digestate. The focus was on the differences in physicochemical properties after 40 and 50 days of composting.

Minimal unpleasant odours were released throughout the composting process, indicating a balanced composting environment without excessive release of N in the form of ammonia gas. Setup A, which consisted solely of chicken manure digestate, transitioned from a highly moist and sludgy texture to a relatively dry, pebble-like structure by day 40. No significant changes in appearance or colour and no maggots or nematodes were observed in this setup throughout the composting period.

Setup B, which was similar to setup A but contained earthworms and garden soil, experienced problems with earthworms escaping and dying as early as day 2, even after several attempts to reset the environment. This experimental control shows that the pure chicken manure digestate was unsuitable for vermicomposting due to its high moisture mass fraction (83.04 %), which created anaerobic conditions unfavourable for earthworms. Therefore, this setup was abandoned and excluded from monitoring after 15 days of composting. It confirmed that the addition of carbonaceous bulking agents is essential for effective vermicomposting as it improves aeration and moisture balance (*16*, *18*, *26*).

**Fig. 1:**
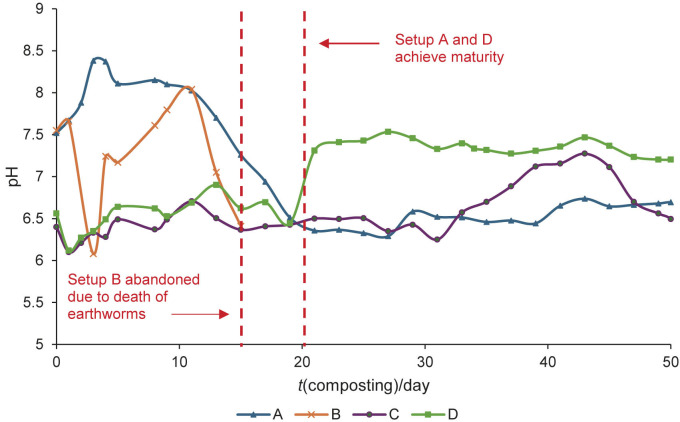
pH profile of compost setups during 50 days of composting. pH values are average of three measurements. Setup: A=pure chicken manure digestate, B=vermicomposted pure chicken manure digestate, C=mixture of chicken manure digestate and kitchen waste and D=vermicomposted mixture of chicken manure digestate and kitchen waste

Setup C, which contained chicken manure digestate and the selected organic additives (spent coffee grounds, bone waste and used kitchen towels), showed a gradual degradation of organic substances, especially the used kitchen towels, which had a distinct appearance throughout the process, as shown in Fig. S1. Maggots and nematodes were observed in the composting medium, feeding on N-dominant materials from day 30, supported by the pH increase after day 30, as shown in Fig. 1 (*26*). Small white spots of undecomposed used kitchen towels were still visible in the setup when samples were taken on day 50. This indicates that the composting process in setup C took longer than in other setups due to the high content of lignocellulosic components in used kitchen towels (*37*).

Setup D had a similar composting composition to setup C, but with the addition of earthworms and garden soil. By day 20, minimal visible feedstock, mainly used kitchen towels, remained, and by the end of the process, the compost had a darker, lumpier texture and an earthy smell, indicating the formation of vermicasting. Towards the end of the process, earthworm activity decreased due to lack of food, which is consistent with the literature data stating that vermicomposting is significantly faster than conventional composting (*17*, *18*, *22*, *26*).

### Temperature and pH profile of composting process

**Fig. 2:**
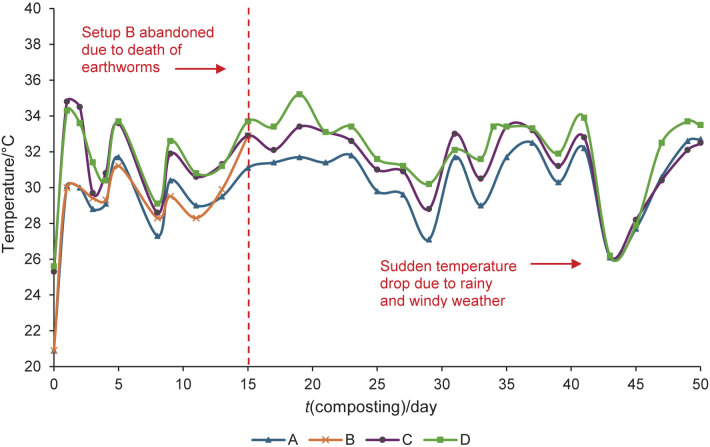
Temperature profile of compost setups during 50 days of composting. Temperature values were average of three measurements. Setup: A=pure chicken manure digestate, B=vermicomposted pure chicken manure digestate, C=mixture of chicken manure digestate and kitchen waste and D=vermicomposted mixture of chicken manure digestate and kitchen waste

Fig. 1 and Fig. 2 show the pH and temperature profiles, respectively, of each compost setup. Three measurements were taken on each sampling day to obtain a mean average value for reporting purposes.

Fig. 1 shows fluctuations in the pH profile that are primarily attributed to microbial and earthworm activity when consuming and degrading organic matter, with weather having only minimal effect (*15*, *26*). Setup A, consisting solely of chicken manure digestate, started with a higher pH due to its initial N mass fraction, which stabilised within the range of 6.0 to 6.5 after day 20. This stabilisation indicates the formation of organic acid through microbial decomposition (*24*, *37*). In setup B, significant fluctuations in pH are observable before the setup was abandoned on day 15, primarily due to decomposition of dead earthworms and subsequent release of proteins, resulting in increased alkalinity (*18*).

In setup C (chicken manure digestate with additives), the pH fluctuated only minimally in the acidic range before day 30 due to the slow decomposition of carbon-containing materials such as used kitchen towels, which are rich in lignocellulosic components, to organic acid and CO_2_ gas (*26*, *38*). A significant increase in pH after day 30 indicated the degradation of proteins and the release of ammonia, which requires a longer composting period for complete maturation (*37*). Nevertheless, the pH of setup C did not stabilise on day 50 when samples were taken, indicating that a longer composting period is needed for maturation. The pH profile for setup D followed a similar trend to setup C, but required a shorter time to reach maturation (20 days), as indicated by the plateau in pH trend after 20 days due to the activity of earthworms, and is consistent with the findings of Zhou *et al.* (*17*) and Azis *et al.* (*37*).

In contrast to the pH profile, the temperature shown in Fig. 2 fluctuated in the range of 26 to 36 °C, without clear distinction of the three characteristic composting phases: the mesophilic phase (25 to 40 °C), the thermophilic or curing phase (>40 °C) and the psychrophilic phase (−10 to 20 °C). These phases were not observed as the composting setups were placed in a shaded outdoor structure, so temperature was only an additional indicator of compost maturation, which is consistent with the study by Shamsuddin *et al.* (*39*). Nevertheless, the temperature range in setup D was relatively higher than in other setups. This can be attributed to the synergistic heat generation by the earthworm and microbial activity, indicating increased decomposition (*22*). This is consistent with the finding of Lew *et al*. (*26*) that vermicomposting often has a higher temperature profile than composting, while the temperature range remains favourable for earthworm living conditions to maintain a complete decomposing activity (*16*).

### Physicochemical and nutrient enrichment of compost

Table 2 compares the nutrient enrichment in NPKC mass fraction, C/N mass ratio and the NPK mass ratio of each compost setup during 40 and 50 days of composting, with the differences (in %) shown as relative enrichment (RE) in Table 3.

**Table 2:** Nutrient enrichment of compost setup during 40 and 50 days of composting

Nutrient element		Setup A	Setup C	Setup D
*w*(C)/%	C_0_	(35.0±1.2)^a^	(44.0±0.6)^b^	(21.7±0.6)^c^
	C_40_	(34.7±3.6)^a^	(39.0±1.9)^a^	(16.5±2.7)^b^
	C_50_	(32.1±0.8)^a^	(32.9±1.5)^a^	(17.4±1.6)^b^
*w*(N)/%	N_0_	(4.87±0.08)^a^	(4.9±1.0)^a^	(2.2±0.1)^b^
	N_40_	(5.4±0.2)^a^	(6.5±0.3)^b^	(4.4±0.2)^c^
	N_50_	(2.48±0.09)^a^	(3.9±0.2)^b^	(1.5±0.3)^c^
*ξ*_C,N_	C_0_/N_0_	(7.2±0.2)^a^	(8.9±2.2)^ab^	(10.1±0.4)^b^
	C_40_/N_40_	(6.4±0.5)^a^	(6.0±0.3)^a^	(3.3±0.5)^b^
	C_50_/N_50_	(13.0±1.0)^a^	(8.4±0.2)^b^	(12.7±0.8)^a^
*w*(P)/%	P_0_	(2.1±0.3)^a^	(1.75±0.04)^b^	(2.38±0.07)^c^
	P_40_	(2.97±0.04)^a^	(1.52±0.04)^b^	(2.70±0.07)^c^
	P_50_	(3.98±0.05)^a^	(4.8±0.3)^b^	(5.36±0.04)^c^
*w*(K)/%	K_0_	(5.1±0.1)^a^	(4.6±0.3)^b^	(5.0±0.1)^a^
	K_40_	(5.3±0.2)^a^	(6.0±0.3)^b^	(6.4±0.3)^b^
	K_50_	(6.8±1.2)^a^	(8.3±1.1)^a^	(9.9±0.7)^b^
*ξ*_N,P,K_	D_0_	2.4:1.0:2.5	2.8:1.0:2.7	1.0:1.1:2.5
	D_40_	1.8:1.0:1.8	4.3:1.0:3.9	1.6:1.0:2.4
	D_50_	1.0:1.6:2.8	1.0:1.2:2.1	1.0:3.6:6.6

Subscripts 0, 40 and 50 denote elemental composition at the beginning, on day 40 and 50 of composting. Mean values in the same row with different letters in superscript differ significantly (p<0.05). Results are expressed as mean value±standard deviation, *N*=3. Setup: A=pure chicken manure digestate, C=mixture of chicken manure digestate and kitchen waste and D=vermicomposted mixture of chicken manure digestate and kitchen waste

**Table 3:** Relative enrichment (RE) of NPKC content after composting

	RE/%		
Nutrient	Setup A	Setup C	Setup D
C_40_	-0.89	-11.33	-24.18
C_50_	-8.12	-25.25	-20.08
N_40_	10.88	32.25	-102.79
N_50_	-49.07	-20.08	-30.23
C_40_/N_40_	-10.58	-32.96	-67.85
C_50_/N_50_	80.50	-6.39	25.12
P_40_	43.48	-13.14	13.44
P_50_	92.27	171.43	125.21
K_40_	3.54	30.42	26.24
K_50_	34.35	82.27	96.22

Positive and negative signs of RE represent the increase and decrease, respectively, in specific elemental composition after composting. Setup: A=pure chicken manure digestate, C=mixture of chicken manure digestate and kitchen waste and D=vermicomposted mixture of chicken manure digestate and kitchen waste

The carbon mass fraction decreased over time in all setups due to the decomposition of organic matter, a typical development during composting (*16*). In setup D, the presence of earthworms minimised carbon loss between days 40 and 50, indicating rapid decomposition in the early stages. In contrast, in setups A and C decomposition of carbon took longer (*17*, *18*). The nitrogen mass fraction increased during the first 40 days in all setups, which can be attributed to nitrogen mineralisation and ammonification processes that increased the ammonia content in the early composting stage (*40*). However, nitrogen mass fraction in setups A and D decreased between days 40 and 50, probably due to nitrogen loss during the formation of oxides or stabilisation of the compost, which is consistent with the increasing trends in the C/N mass ratio (*16*). This observation was further supported by the results of one-way ANOVA for the C/N mass ratio between setups A and D on day 50 of composting. While a lower C/N mass ratio indicates the maturity of the compost, nitrogen release stabilises the mass ratio in the optimal range of 10:1 to 15:1, which is consistent with previous studies (*25*, *26*, *28*).

The phosphorus mass fraction increased in all setups, with vermicomposting (setup D) showing the most significant increase. This was due to phosphorus-solubilising microorganisms and the conversion of organic phosphorus into plant-available inorganic forms when organic matter passed through the earthworms’ guts (*41*). Similarly, potassium mass fraction increased in all setups, with vermicompost reaching the highest values due to high microbial activity that solubilised insoluble potassium compounds (*25*). These results were supported by the results of one-way ANOVA, which show that setup D was significantly different from setups A and C after 50 days of composting.

Comparing the initial NPK mass ratio of the fresh chicken manure digestate (2.35:1:2.45), all compost setups had lower nitrogen mass ratio but significantly higher phosphorus and potassium mass ratios, especially in the vermicompost setup (1:3.57:6.58). This increase in NPK content during vermicomposting was attributed to the mineralisation of nutrients, with previous studies emphasising the slow release of nutrients in vermicompost, reducing the environmental pollution from nutrient leaching (*21*, *42*).

The mass yield and moisture mass fraction of the final compost product from each setup are summarised in Table 4. The mass was reduced in all setups, with setup A having the lowest value due to its high moisture mass fraction, which created anaerobic conditions that were unfavourable for microbial activity (*26*). In contrast, due to the healthier aerobic conditions and higher initial nitrogen-to-carbon mass ratio, decomposition of organic matter in setup C was faster, which resulted in higher mass loss (*28*). Setup D had the highest mass reduction, which was attributed to the synergistic effects of earthworms and microorganisms as strong decomposers that degraded rigid carbon-rich materials and broke down waste, resulting in higher mass reduction than setup A (*22*).

**Table 4:** Mass yield, initial and final moisture mass fraction of each compost setup

Compost	*m*_final_/kg	*m*_2_/kg	*w*(MC_1_)/%	*w*(MC_2_)/%	*Y*/%	*m*_reduction_/%
A	0.71	0.71	(83.0±0.3)^a^	(67.1±0.4)^a^	91.76	8.23
C	0.63	0.63	(68.0±0.1)^b^	(67.6±0.2)^a^	48.01	51.99
D	2.33	0.83	(46.1±0.2)^c^	(48.9±0.7)^b^	44.89	55.11

Mean values in the same column with different letters in superscript differ significantly (p<0.05). Results are expressed as mean value±standard deviation, *N*=3. *m*_final_=final mass of the compost (including gardening soil), MC_1_ and MC_2_=initial and final moisture of compost mixture, respectively, *m*_2_=difference between *m*_final_ and mass of soil, *m*_soil_. Setup: A=pure chicken manure digestate, C=mixture of chicken manure digestate and kitchen waste and D=vermicomposted mixture of chicken manure digestate and kitchen waste

## CONCLUSIONS

Organic additives, including spent coffee grounds, bone waste, banana peels and used kitchen towels, were characterised. The results showed that spent coffee grounds had the highest carbon (50.05 %) and potassium (6.54 %) mass fraction, bone waste had the highest nitrogen (5.74 %) and phosphorus (22.26 %) mass fraction, and used kitchen towels had the highest C/N mass ratio (15.51:1). A comprehensive analysis of the four composting and vermicomposting setups showed that the vermicomposting setup with organic additives (setup D) resulted in the highest improvement in nutrients with an NPK mass ratio of 1:3.57:6.58 on day 50 compared to the initial NPK mass ratio of 2.35:1:2.45 for chicken manure digestate. Notably, setup D reached maturity in the shortest composting time (20 days), with a significant mass reduction of 54.22 % compared to the initial feedstock. These results confirm the effectiveness of vermicomposting with organic kitchen waste in improving the physicochemical properties of chicken manure digestate, while achieving substantial mass reduction of organic waste in a shorter composting time. Several sustainable development goals (SDGs) have been reached by promoting circular economy with a cost-effective and easy-to-implement solution of vermicomposting for organic waste management with significant environmental and economic benefits.

## Appendix

**Table S1:** Composition of compost setups

	*m*/kg			
Setup	Chicken manure digestate	Spent coffee grounds	Bone waste	Used kitchen towels
A	1.50	-	-	-
B	1.50	-	-	-
C	0.50	0.40	0.05	0.45
D	0.50	0.40	0.05	0.45

Setup: A=pure chicken manure digestate, B=vermicomposted pure chicken manure digestate, C=mixture of chicken manure digestate and kitchen waste and D=vermicomposted mixture of chicken manure digestate and kitchen waste
